# YTHDC1 regulates distinct post-integration steps of HIV-1 replication and is important for viral infectivity

**DOI:** 10.1186/s12977-022-00589-1

**Published:** 2022-01-31

**Authors:** Sarah N’Da Konan, Emmanuel Ségéral, Fabienne Bejjani, Maryam Bendoumou, Mélissa Ait Said, Sarah Gallois-Montbrun, Stéphane Emiliani

**Affiliations:** 1 Institut Cochin, INSERM, CNRS, Université de Paris, 75014 Paris, France; 2 Service of Molecular Virology, Department of Molecular Biology, Université Libre de Bruxelles, 6041 Gosselies, Belgium

**Keywords:** HIV-1, Epitranscriptomic, m^6^A methylation, METTL3/14, YTHDF, YTHDC1, RNA biogenesis

## Abstract

**Background:**

The recent discovery of the role of m^6^A methylation in the regulation of HIV-1 replication unveiled a novel layer of regulation for HIV gene expression. This epitranscriptomic modification of HIV-1 RNAs is under the dynamic control of specific writers and erasers. In addition, cytoplasmic readers of the m^6^A mark are recruited to the modified viral RNAs and regulate HIV-1 replication. Yet, little is known about the effects of m^6^A writers and readers on the biogenesis of HIV-1 RNAs.

**Results:**

We showed that the METTL3/14 m^6^A methyltransferase complex and the m^6^A YTHDF2 cytoplasmic reader down regulates the abundance of HIV-1 RNAs in infected cells. We also identified the m^6^A nuclear reader YTHDC1 as a novel regulator of HIV-1 transcripts. In HIV-1 producer cells, we showed that knocking down YTHDC1 increases the levels of unspliced and incompletely spliced HIV-1 RNAs, while levels of multiply spliced transcripts remained unaffected. In addition, we observed that depletion of YTHDC1 has no effect on the nuclear cytoplasmic distribution of viral transcripts. YTHDC1 binds specifically to HIV-1 transcripts in a METTL3-dependent manner. Knocking down YTHDC1 reduces the expression of Env and Vpu viral proteins in producer cells and leads to the incorporation of unprocessed Env gp160 in virus particles, resulting in the decrease of their infectivity.

**Conclusions:**

Our findings indicate that, by controlling HIV-1 RNA biogenesis and protein expression, the m^6^A nuclear reader YTHDC1 is required for efficient production of infectious viral particles.

**Graphical Abstract:**

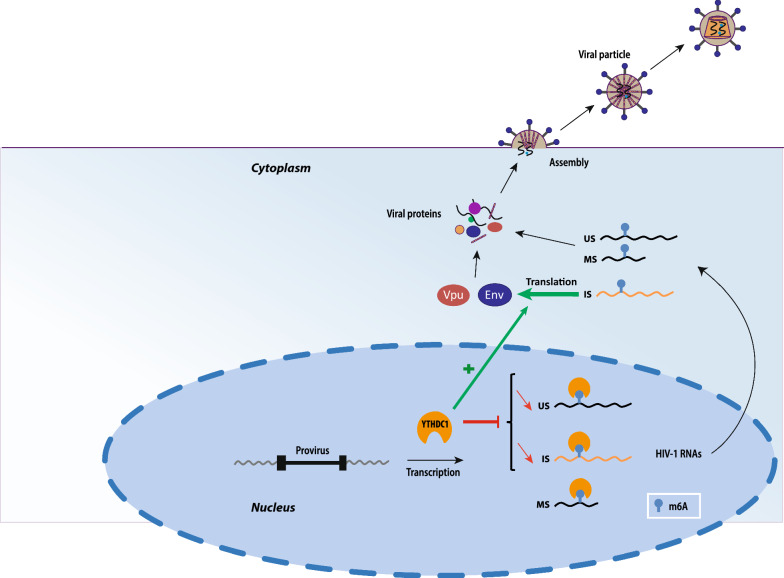

**Supplementary Information:**

The online version contains supplementary material available at 10.1186/s12977-022-00589-1.

## Background

Cellular RNAs are post-transcriptionally subjected to more than one hundred chemical modifications that contribute to their biogenesis and function. Among them, *N*^6^-methyladenosine (m^6^A) is the most abundant modification in eukaryotic mRNA that takes place at the RRm^6^ACH consensus motif (R = G/A, H = U/A/C) and is found in at least 25% of the mRNAs [1, 2]. The core of the methyltransferase complex responsible for the methylation of *N*^*6*^*-*adenosine is composed of the heterodimer Methyltransferase-like 3 (METTL3) and METTL14. Within this complex, METTL3 is the catalytically active subunit while METTL14 plays a structural role and is involved in RNA recognition [3–5]. Additional subunits including WTAP, VIRMA, RBM15/15B, ZC3H13 and HAKAI bind to the core METTL3/14 to form a larger holocomplex which regulates METTL3/14 recruitment to RNA and its activity [6–10]. The m^6^A modification is dynamic and can be removed by two demethylases, also called erasers: FTO and ALKBH5 [11, 12]. m^6^A methylation regulates different steps of RNA biogenesis including stability, splicing, nuclear export and translation [13]. The deposition of the m^6^A mark can directly promote the recruitment of readers involved in the regulation of RNA biogenesis and translation. Several proteins from the YTH family have been identified as m^6^A readers including the 3 cytosolic paralogs YTHDF1, YTHDF2, YTHDF3, the nuclear YTHDC1 and the cytosolic YTHDC2 [14]. YTH proteins contain a hydrophobic aromatic cage within the YTH domain that binds m^6^A modified RNA [13–20]. METTL3/14-mediated m^6^A deposition promotes mRNA degradation and translation via the binding of YTHDF readers [21–23]. In addition, the nuclear reader YTHDC1, which has been shown to regulate splicing, alternative polyadenylation and nuclear export also decreases the abundance of cellular transcripts [24–26].

Recently, m^6^A modifications were identified on HIV-1 transcripts, at a level slightly higher to the one observed on cellular mRNAs, and were shown to affect viral replication [27–29]. Beside the differences between the m^6^A peaks identified by these three studies, which are likely due to the different m^6^A mapping techniques used, several overlapping m^6^A clusters were identified within the 3’ UTR region of the viral genome. Moreover, these m^6^A clusters partially, but not completely, overlap with the binding sites of all 3 YTHDF readers [27, 29].

While m^6^A methylation was shown to affect HIV-1 replication, the molecular mechanisms by which m^6^A controls HIV-1 infection remain to be understood [27–30]. Several steps of HIV-1 life cycle were identified to be regulated by m^6^A methylation. In virus producing cells, two studies have shown that knocking down the METTL3/14 complex reduces HIV-1 Gag protein expression, indicating that m^6^A is required for viral expression [28, 29]. However, studies of the role of YTHDF readers on HIV-1 replication have reached opposing conclusions. In target cells, YTHDF readers were found to inhibit early steps of HIV-1 replication (e.g. reverse transcription) by directly interacting with incoming viral gRNA [29, 30]. However, in virus producing cells, all 3 YTHDF readers are promoting HIV-1 protein expression [27]. In addition, it has been reported that the YTHDF3 reader is directly incorporated within the virion via its interaction with the nucleocapsid, and subsequently cleaved inside the particle by the viral protease [31]. Yet, the presence of YTHDF readers in viral particles was not observed in another study [30].

As m^6^A methylation directly affects RNA destabilization, splicing, cellular localization and translation, we decided to explore the effect of m^6^A writers and readers on the fate of the 3 classes of HIV-1 transcripts. Here, we report that knocking down subunits of the METTL3/14 holocomplex resulted in an increase of all 3 classes of HIV-1 RNAs. Similarly, depletion of the YTHDF2, but not YTHDF1 or YTHDF3, increased the levels of HIV-1 transcripts in a single round infection assay. Interestingly, in HIV-1 producer cells, we found that the m^6^A nuclear reader YTHDC1 decreases the abundance of HIV-1 unspliced (US) and incompletely spliced (IS) but not multiply spliced (MS) RNAs. Knocking down YTHDC1 does not affect the cellular localization of HIV-1 RNAs. Furthermore, YTHDC1 directly interacts with HIV-1 RNAs in a METTL3-dependent manner. Interestingly, we found that YTHDC1 controls HIV-1 Vpu and Env protein expression and the infectivity of viral particles. Together, our data suggest that YTHDC1 regulates the fate of HIV-1 RNAs at distinct steps of their biogenesis.

## Results

### Knockdown of the m^6^A METTL3/14 writer complex and the YTHDF2 reader upregulates HIV-1 mRNA levels in infected cells

**Fig. 1:**
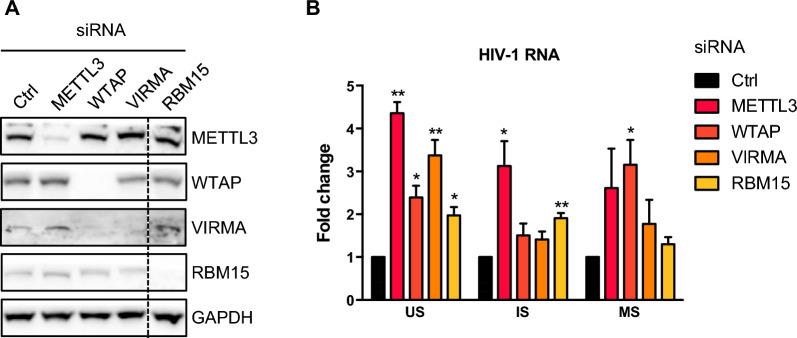
Depletion of m^6^A writer complex subunits increase HIV-1 mRNA levels in infected cells. **A** HeLa cells were transfected with a control (Ctrl) or METTL3, WTAP, VIRMA and RBM15-targeting siRNAs, as indicated. Cellular proteins knockdown was confirmed by western blot analysis of cell lysates transfected with indicated siRNAs. **B** 3 days after siRNA (targeting RBM15) or 4 days after siRNA transfection (targeting METTL3, WTAP and VIRMA), cells were infected with a single round VSVg-pseudotyped HIV-1 virus. 24 h.p.i., the relative abundance of unspliced (US), incompletely spliced (IS) and multiply spliced (MS) HIV-1 RNAs was monitored by RT-qPCR. Results are expressed in fold change over the control siRNA (Ctrl). Data are presented as mean ± S.D. (*n* = 3). *P* values were calculated using one sample t-test (*p < 0.05, **p < 0.001)

Previous studies have shown that the m^6^A methyltransferase complex regulates HIV-1 replication. In particular, both METTL3 and METTL14 were shown to promote HIV-1 replication in multiple round infection assays and to be required for efficient Gag protein expression in cells transfected with an HIV-1 proviral plasmid [28, 29]. However, the direct effect of the m^6^A methyltransferase complex on HIV-1 RNA levels in a single round infection assay remains to be explored. Thus, we first knocked down METTL3, WTAP, VIRMA and RBM15 protein expression in HeLa cells. While siRNAs against METTL3, VIRMA and RBM15 specifically silenced their respective target, we noticed that silencing WTAP also inhibited VIRMA expression (Fig. 1A). Next, HeLa cells were infected with a VSVg-pseudotyped NL4-3 virus and newly synthesized HIV-1 RNAs were measured by RT-qPCR using primers specific for US, IS and MS HIV-1 transcripts (Additional file 1: Fig. S1). We observed that US transcripts levels increased by 2- to fourfold upon depletion of METTL3, WTAP, VIRMA and RBM15 while an increase of IS and MS mRNA levels was only seen upon depletion of some of the subunits (Fig. 1B). These results indicate that depletion of components of the m^6^A methyltransferase complex up regulates HIV-1 RNA abundance in infected cells.

Recent reports demonstrated that the cytoplasmic m^6^A reader paralogs YTHDF 1 to 3 are recruited to m^6^A residues within the HIV-1 RNA genome, yet their impact on HIV replication has led to opposite results [27, 29, 30]. While the three YTHDF paralogs were identified as positive regulators of both HIV-1 RNA and protein expression [27], other studies showed that the YTHDF readers could inhibit both early and late steps of HIV-1 replication [29, 30]. Thus, we sought to determine the effect of YTHDF readers on HIV-1 RNA levels using the same single round infection assay. We knocked down each of the YTHDF paralogs using specific siRNA and quantified their respective mRNA levels by RT-qPCR. A robust decrease in RNA expression was observed for each YTHDF paralog upon its specific siRNA transfection. Interestingly, we also observed compensatory effects at the RNA levels for specific siRNAs on other YTHDF paralogs (Additional file 1: Fig. S2A). Inhibition of YTHDF readers expression was confirmed by western blotting (Additional file 1: Fig. S2B). Next cells were infected with a VSVg-pseudotyped NL4-3 virus and HIV-1 RNA was measured by RT-qPCR using primers specific for US, IS and MS viral transcripts. The effect was most marked for YTHDF2 knock down, resulting in an increase of all three classes of HIV-1 RNA, while YTHDF1 and YTHDF3 depletion only modestly impacted HIV-1 transcripts abundance (Additional file 1: Fig. S2C). Thus, our results indicate that YTHDF2, but not YTHDF1 and YTHDF3, decreases HIV-1 RNA abundance in a single round infection assay. However, we cannot exclude that by increasing YTHDF2 expression, the knock down of YTHDF1 and YTHDF3 could damper the actual effect of these two readers on HIV RNA levels.

### Knockdown of YTHDC1 upregulates HIV-1 transcripts levels without affecting their cellular localization

**Fig. 2:**
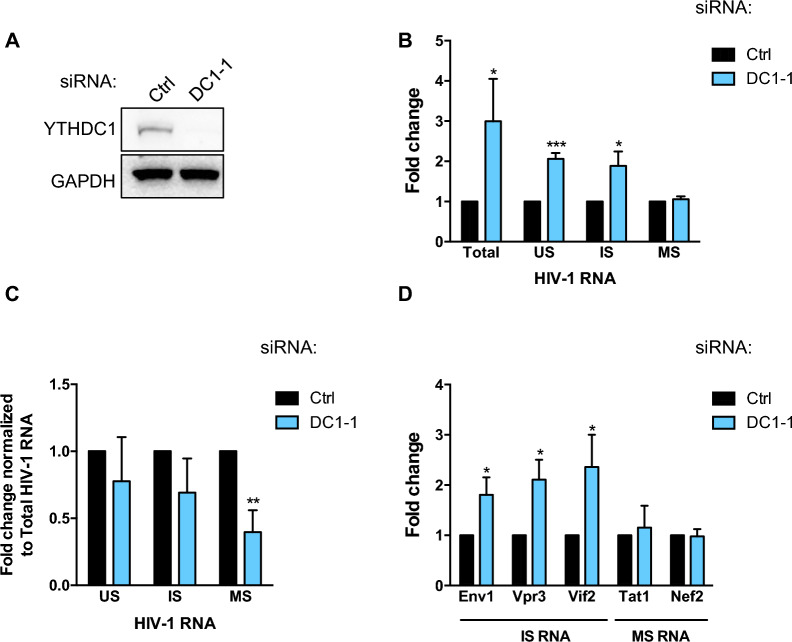
Downregulation of YTHDC1 increases US and IS viral RNAs without affecting the abundance of MS transcripts. **A** HeLa/LAV cells were transfected with a control (Ctrl) or YTHDC1-targeting siRNA (DC1-1). 3 days later, YTHDC1 knock down was confirmed by western blot. **B** The relative abundance of Total, US, IS and MS HIV-1 RNAs was monitored by RT-qPCR. **C** Levels of US, IS and MS RNAs from (**B**) were normalized to the level of Total viral RNA. **D** The relative abundance of IS RNAs, Env1, Vpr3 and Vif2, an MS RNAs Tat1 and Nef2 was monitored by RT-qPCR. Results are expressed in fold change over the control siRNA (Ctrl). Data are presented as mean ± S.D. (*n* = 4). *P* values were calculated using one sample t-test (*p < 0.05, **p < 0.001, ***p < 0.0001)

HIV-1 utilises alternative splicing to generate more than 50 different viral transcripts from a single pre-mRNA [32]. HIV-1 alternative splicing takes place post-transcriptionally, which implies that the US precursor will generate IS RNAs, which themselves will generate MS RNAs [33, 34]. As YTHDC1 is a nuclear reader that was recently described to regulate splicing of cellular mRNA, we sought to study the role of YTHDC1 in the regulation of HIV-1 RNAs [24]. To bypass any potential effect of YTHDC1 depletion on the early steps of HIV-1 replication (e.g. reverse transcription and/or integration), we performed experiments in HeLa/LAV cells that carry copies of integrated proviruses expressing high levels of viral proteins [35, 36]. In these cells, new rounds of infection do not occur due to the lack of CD4 receptor expression at the cell surface ([37] and data not shown). Thus, knocking down YTHDC1 in these cells will solely affect the post-integration steps of viral replication, from transcription to particle release. HeLa/LAV cells were transfected with a control siRNA (Ctrl) or a siRNA targeting YTHDC1 (DC1-1) (Fig. 2A), then 72 h later cells were harvested and we analyzed Total, US, IS and MS viral RNAs by quantitative real-time RT-PCR using sets of specific primers. The level of each mRNA species was normalized to the level of GAPDH mRNA. An approximately 2 to threefold increase in Total, US and IS HIV-1 RNA levels was observed upon YTHDC1 depletion while the MS transcripts abundance was not affected by the depletion of YTHDC1 (Fig. 2B). When relative levels of US, IS and MS transcripts were normalized to the level of Total HIV-1 RNA, we observed a significant decrease of MS RNAs (Fig. 2C). The relative abundance of viral isoforms from the IS and MS transcripts was further quantified. Env1, Vpr3 and Vif2, all belonging to the IS class of transcripts, were increased by 1.8 to 2.3-fold upon YTHDC1 depletion (Fig. 2D). Again, the MS isoforms Tat1 and Nef2 were not significantly affected by YTHDC1 knock down (Fig. 2D). Thus, our observations indicate that the depletion of YTHDC1 increases US and IS viral RNAs without affecting the abundance of MS transcripts.

**Fig. 3:**
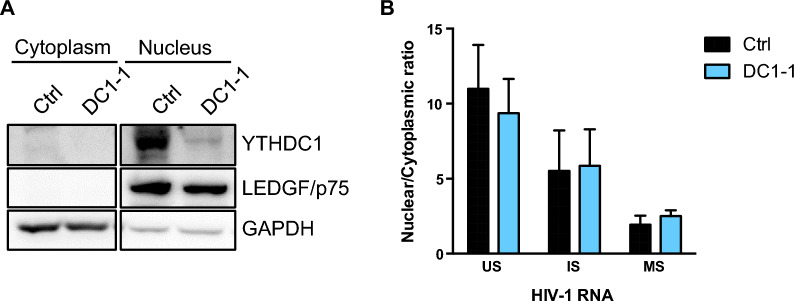
The subcellular distribution of HIV-1 RNAs is not affected by YTHDC1 knockdown. **A** HeLa/LAV cells were transfected with a control (Ctrl) or YTHDC1-targeting siRNA (DC1-1). 3 days later, cells were fractionated and the purity of the fractions was assessed by western blot using antibodies against the nuclear protein LEDGF/p75 and the cytoplasmic protein GAPDH. Low level of nuclear contamination by cytoplasmic GAPDH can be observed. YTHDC1 depletion was also monitored using an anti-YTHDC1 antibody. **B** The relative abundance of US, IS and MS HIV-1 RNAs in the nucleus and in the cytoplasm was monitored by RT-qPCR, and the nuclear to cytoplasmic ratio of HIV-1 RNAs was represented. Data are presented as mean ± S.D. (*n* = 4). *P* values calculated using paired t-test were not significant

In addition to its role in splicing, YTHDC1 was recently described to mediate the transport of cellular mRNA from the nucleus to the cytoplasm leading us to analyze the effect of YTHDC1 knock down on the level of HIV-1 RNAs within this cellular compartments [25]. HeLa/LAV cells were first knocked down for YTHDC1 expression. We then isolated nuclear and cytoplasmic fractions and extracted RNA to quantify the levels of US, IS and MS viral transcripts. To control the quality of the fractionation process, we first confirmed by Western blotting the enrichment of YTHDC1 and chromatin reader LEDGF/p75 proteins within the nuclear fraction. We also checked that GAPDH was mostly present in the cytoplasmic fraction (Fig. 3A). The distribution of viral RNAs between the nucleus and cytoplasm was not affected by YTHDC1 knock down (Fig. 3B), indicating that the nuclear reader is not involved in the nuclear export of HIV-1 transcripts.

### YTHDC1 binds HIV-1 transcripts in a METLL3-dependent manner

**Fig. 4:**
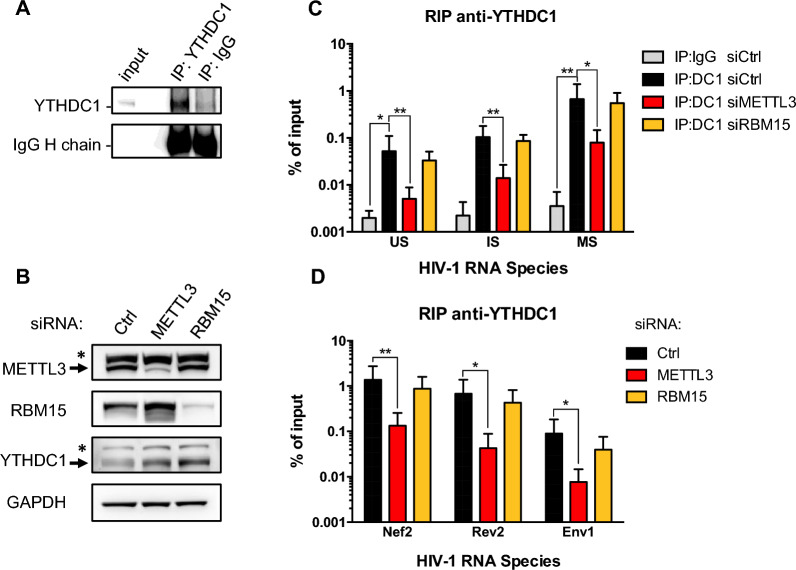
YTHDC1 interacts with the 3 classes of HIV-1 mRNAs in a METTL3-dependent manner. **A** Western blot showing that endogenous YTHDC1 was specifically immunoprecipitated from HeLa/LAV cells. IgGs were used as control immunoprecipitation. **B** HeLa/LAV cells were transfected with control (Ctrl) or METTL3 and RBM15-targeting siRNA and 3 days later, knock down of METTL3 and RBM15 was confirmed by western blot. **C** YTHDC1 was immunoprecipitated from HeLa/LAV cells transfected in (**B**) and levels of YTHDC1-associated US, IS and MS viral RNAs were quantified by RT-qPCR. Data are presented as mean ± S.D. (*n* = 4). *P* values were calculated using paired t-test (*p < 0.05, **p < 0.01). **D** Same as (**C**), except the relative abundance of MS RNAs Tat1 and Rev2 and IS RNA Env1 was monitored by RT-qPCR. Results are expressed as percentage of input. Data are presented as mean ± S.D. (*n* = 3). *P* values were calculated using paired t-test (*p < 0.05, **p < 0.01)

While previous reports have shown that YTHDF paralogs are recruited to the HIV-1 RNA genome [27, 29], an interaction between YTHDC1 and viral transcripts has not been described yet. In addition, it is not known whether YTH readers could distinguish between specific classes of HIV-1 transcripts. Our observations on the effect of YTHDF2 and YTHDC1 on HIV-1 RNAs abundance led us to examine whether these two m^6^A readers could specifically interacts with HIV-1 transcripts using RNA immunoprecipitation (RIP) assay. Thus, to analyze whether endogenous YTHDC1 can bind HIV-1 transcripts in HeLa/LAV cells in a METTL3 complex-dependent manner, RIP experiments were performed in cells knocked down for two components of the writer complex: the catalytic subunit METTL3 and RBM15, an associated protein involved in m^6^A methylation site selection (Fig. 4A, B). We observed that YTHDC1 binds to the 3 classes of HIV-1 RNA with a preference for MS transcripts (Fig. 4C). In sharp contrast, in HeLa cells infected with a VSVg-pseudotyped NL4-3 virus, YTHDF2 interacts preferentially with US transcripts and to a lesser extend to MS RNAs (Additional file 1: Fig. S3). Knocking down METTL3 expression significantly reduced the binding of YTHDC1 to US, IS and MS HIV-1 RNAs, suggesting that the m^6^A methylation complex promotes YTHDC1 binding to viral transcripts. These interactions were not impaired upon RBM15 depletion, indicating that this subunit is not involved in the selection and/or recognition of YTHDC1 binding sites on HIV-1 RNA (Fig. 4C). Similar effects were observed when primers specific for the MS transcripts Nef2, Rev2 and the IS transcript Env1 were used (Fig. 4D). Thus, our data indicate that YTHDC1 recruits the 3 classes of HIV-1 RNAs, with a preference in MS versus IS and US transcripts. Importantly, YTHDC1 HIV-1 RNA-binding is dependent on the catalytic subunit METTL3 but not on the regulatory subunit RBM15.

### YTHDC1 depletion affects Vpu and Env expression levels in infected cells

**Fig. 5:**
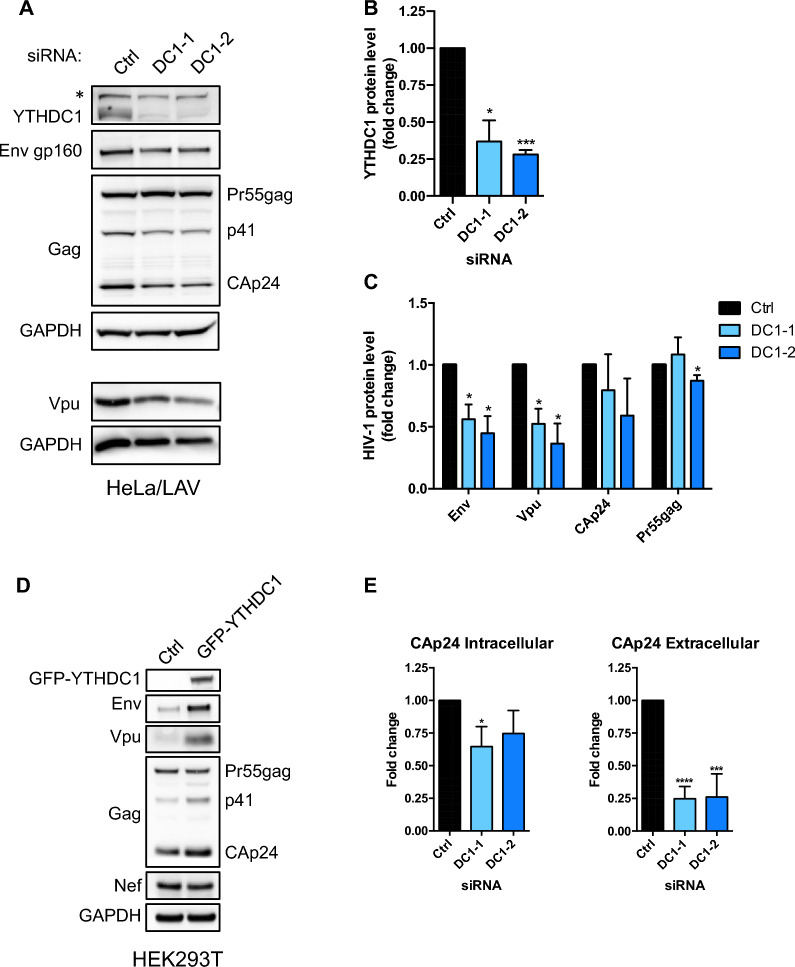
YTHDC1 depletion affects Env and Vpu expression in infected cells. HeLa/LAV cells were transfected with control (Ctrl) or 2 distinct siRNAs targeting YHTDC1 (DC1-1 and DC1-2) and 72 h later, cells were harvested and **A** HIV-1 viral proteins Env, Gag and Vpu, YTHDC1 and GAPDH were analyzed by western blot (*, nonspecific band) and **B** YTHDC1 and **C** HIV-1 proteins band intensities were quantified by phosphoimaging and normalized to GAPDH (*n* = 3). Fold change of protein levels upon DC1 knock down over the Ctrl are plotted. Data are presented as mean ± S.D. (*n* = 3). *P* values were calculated using one sample t-test (*p < 0.05, ***p < 0.001). **D** HEK293T cells were co-transfected with a control plasmid or a plasmid expressing GFP-YTHDC1 and pNL4-3. 48 h. later cells harvested and cell lysates were analyzed by western blotting for HIV-1 viral proteins Env, Gag and Vpu and Nef. **E** ELISA quantification of CAp24 in cell lysates (CAp24 Intracellular; *n* = 4) and released into the cell supernatants (CAp24 Extracellular; *n* = 6). Data are represented as mean ± S.D. *P* values were calculated using one sample t-test (***p < 0.001, ****p < 0.0001)

To further explore the role of YTHDC1 on HIV-1 expression, HeLa/LAV cells were transfected with control siRNA (Ctrl) and two distinct siRNAs targeting YTHDC1 (DC1-1 and DC1-2) and 3 days later viral protein expression was quantified. YTHDC1 protein expression was robustly decreased by more than threefold in siRNA DC1-1 and DC1-2 treated cells (Fig. 5A, B). Interestingly, this decrease led to a significant reduction of Env gp160 (44 to 55%, upon treatment with siRNA DC1-1 and DC1-2, respectively) and Vpu protein expression (48 to 64%, upon treatment with siRNA DC1-1 and DC1-2, respectively) (Fig. 5A, C). In contrast, little or no effect was observed on the levels of the Pr55gag precursor, suggesting that YTHDC1 might differently affect HIV proteins expression (Fig. 5A, C). A sharp reduction of Env and Vpu expression was also observed in YTHDC1-depleted HeLa cells transiently expressing HIV-1 (Additional file 1: Fig. S4A and B). Mirroring this effect, overexpression of GFP-YTHDC1 strongly increased Env and Vpu protein expression in HEK293T cells (Fig. 5D). CAp24 ELISA quantification yielded a 25 to 35% decrease of CAp24 in HeLa/LAV lysates treated with siRNA against YTHDC1 (Fig. 5E). This decrease in intracellular CAp24 is also associated with a fourfold decrease in extracellular CAp24 reflecting a sharp reduction of released virus upon YTHDC1 depletion (Fig. 5E).

### YTHDC1 depletion affects Env processing and incorporation into viral particles

**Fig. 6:**
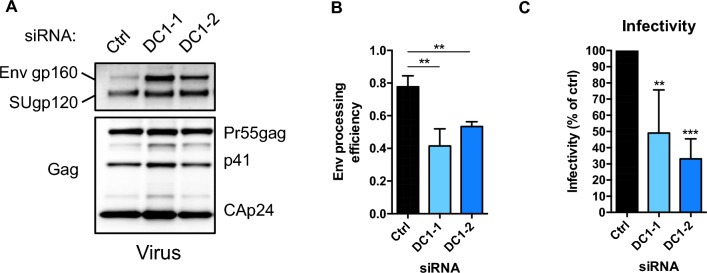
YTHDC1 depletion affects the incorporation of mature Env into viral particles. **A** HIV-1 viral proteins Env and Gag in cells supernatants were analyzed by western blot. Equal amounts of CAp24 were loaded per lane to assess Env content per fixed amounts of viral particles. **B** Env signals were quantified and values obtained were used to calculate the Env processing efficiency (ratio of the level of processed SUgp120 relative to the level of total Env (Env gp160 + SUgp120)). Data are presented as mean ± S.D. (*n* = 3). *P* values were calculated using unpaired t-test (**p < 0.01). **C** Infectivity of virions was measured by HeLa TZM-bl reporter assay and normalized to the quantity of CAp24 in the supernatants. Values are presented as means ± SD (*n* = 6). *P* values were calculated using one sample t-test (*p < 0.05, ***p < 0.001)

To further characterize the effect of YTHDC1 depletion on the composition of viral particles, we analyzed Gag and Env by western blot loaded with equivalent amounts of CAp24 (Fig. 6A). Interestingly, we observed that the depletion of YTHDC1 resulted in higher levels of the amount of total amount of Env (SUgp120 + Env gp160 Env) incorporated into virions, while the processing efficiency on Env (as determined by the ratio of SUgp120/(SUgp120 + Env gp160)) was reduced (Fig. 6B). To further assess the effect of YTHDC1 on the processing of the Env precursor, we measured the infectivity of released virions by calculating the level of infected reporter cells normalized by the level of CAp24 for each viral production. Knocking down YTHDC1 in HeLa/LAV producing cells reduced the infectivity of virions by 2- to threefold (Fig. 6C). Altogether, these results indicate that YTHDC1 stimulates the expression of Env and Vpu HIV-1 proteins in infected cells. Furthermore, YTHDC1 silencing favors the incorporation of Env precursor gp160 into virions and impairs the infectivity of released viral particles.

## Discussion

In this study, we have sought to analyze the effect of m^6^A methylation writers and readers on the fate of HIV-1 RNAs and their impact on infection. Using a single round infection assay, we first showed that subunits of the methyltransferase complex and the cytoplasmic reader YTHDF2 down regulate the levels of HIV RNAs (Fig. 1 and Additional file 1: Fig. S2). These results are consistent with previously reported effects of m^6^A-dependant regulation of the stability of cellular RNAs by the m^6^A cytoplasmic readers [22, 38]. In particular, YTHDF2 has been shown to recognize and to promote degradation of m^6^A-modified RNAs via two independent pathways: the CCR4-NOT deadenylase complex and the RNAse P/MRP complex [39, 40]. Contrary to what we observed for YTHDF2, YTHDF1 and YTHDF3 knock down had not effect on HIV-1 RNAs abundance (Additional file 1: Fig. S2). This is likely due to compensatory effects between YTHDF paralogs [38]. Indeed, we noticed that the depletion of YTHDF1 and YTHDF3 increased YTHDF2 RNA level, while knocking down YTHDF2 stimulates YTHDF1 RNA level (Additional file 1: Fig. S2). However, our results conflict with a previous study showed that overexpression of YTHDF paralogs in 293 T cells increased US and MS HIV-1 RNAs in a single round infection assay [27]. The different approaches used in the two studies could account for these opposite observations. Indeed, it has been shown that upon overexpression, YTHDF proteins tend to aggregate into membrane-less compartments resembling stress granule structures via their low-complexity domains [38, 41, 42]. Relocation to YTHDF-induced stress granule-like structures could alter the fate of m^6^A-modified viral mRNAs, for example by protecting them from degradation as it has been shown after heat shock [39, 40].

Importantly, our study identified the nuclear m^6^A reader YTHDC1 as a novel regulator of the HIV-1 RNAs fate. YTHDC1 has been described to regulate stability, splicing, alternative polyadenylation and nuclear export of m^6^A modified cellular and viral transcripts [25, 26, 43–45]. HIV-1 splicing is not co-transcriptional as a full-length US transcript of 9.2 kb must be first fully synthesized to be subsequently spliced into IS transcripts of 4 kb, from splice donor D1 to splice acceptors A1, A2, A4 and A5, which can be further spliced into MS transcripts of 2 kb through an additional splicing event between D4 and A7 [32]. Surprisingly, while we clearly see that the depletion of YTHDC1 increases by twofold the levels of US and IS transcripts, we did not observe a variation in the abundance of MS RNAs, indicating that this class of transcripts is regulated separately. One hypothesis is that YTHDC1 could specifically bind to m^6^A-dependent motifs potentially involved in RNA decay that are only present in US and IS RNAs and selectively down regulates their abundance without impacting MS transcripts. Another possibility could be that YTHDC1 is down regulating all 3 classes of HIV-1 transcripts but is also having an additional effect on the stimulation of the D4 to A7 splice. In this case, knocking down YTHDC1 would increase US and IS RNAs, but not MS RNAs due to a subsequent block of IS to MS splice. Interestingly, our RIP experiments showing that YTHDC1 preferentially interacts with MS transcripts could further support a role of YTHDC1 in alternative splicing (Fig. 4 and see “Discussion”).

As a role of YTHDC1 in nuclear export of cellular m^6^A-modified transcripts has been previously reported, we analysed the effect of YTHDC1 depletion on the cellular distribution of viral RNAs [25]. HIV-1 US and IS transcripts are directly interacting with the viral Rev protein via their RRE element present within the D4-A7 exon [32]. Rev binding to RRE promotes the nuclear export of these intron-containing viral transcripts that are otherwise retained within the nucleus, while fully spliced HIV-1 transcripts are exported through the NXF1-export pathway. It has been proposed that m^6^A methylation of adenosines within the Rev-responsive element (RRE) region could directly control Rev-dependent nuclear export of HIV-1 US and IS transcripts [28]. However, the presence of m^6^A modifications in the RRE region has not been confirmed by other studies [27, 28, 46, 47]. As we observed an increase in US and IS transcripts upon YTHDC1 depletion, we resonated that a defect in nuclear export could lead to their accumulation in the nucleus. However, our cellular fractionation experiments showed that knocking down YTHDC1 did not affect the nuclear/cytoplasmic distribution of the 3 classes of viral transcripts, ruling out a role of YTHDC1 in HIV-1 RNAs nuclear export (Fig. 3).

Interestingly, we were able to show that YTHDC1 binds to the 3 classes of viral transcripts with a higher enrichment for MS RNAs (Fig. 4). As mentioned above, a specific interaction with MS transcripts could reflect the ability of YTHDC1 to specifically interact and/or regulate with the D4/A7 splice junction. Importantly, these interactions are dependent on METTL3 expression but not RBM15, a cofactor that recruits the m^6^A methyltransferase complex to specific sites [8]. These data suggest that YTHDC1 recruitment to viral RNAs is m^6^A dependent. On the other hand, YTHDF2 favours interaction with US transcripts and to a lower extend MS transcripts, while IS transcripts were not enriched in our RIP assay. How the different YTH readers could distinguish between the 3 classes of HIV-1 RNAs it is not yet understood. m^6^A sites were mapped on the HIV-1 RNA genome by several groups and despite discrepancies likely due to the different approaches used, enrichment of the m^6^A marks within the 3’- UTR region shared by the 3 classes of viral transcripts was detected in each study [27–29]. However, the number and distribution of m^6^A sites in the different HIV-1 mRNA isoforms is still unknown and will be technically challenging to determine. US and IS HIV-1 RNAs contain retained introns that could potentially be methylated. Yet, the detection of m^6^A sites in HIV-1 introns is inconsistent between the different studies [27–29, 47]. Thus, evidence of the enrichment of the m^6^A mark in specific HIV-1 RNA isoforms that could explain the specific binding of YTH readers are still lacking. We recently used the long-reads sequencing technology to identify and to quantify HIV-1 isoforms [34]. This sequencing approach applied directly to RNA in combination with m^6^A meRIP was recently used to identify m^6^A marks on adenovirus transcripts [45]. A similar approach could help to resolve the m^6^A viral transcriptome mapping of m^6^A marks.

Importantly, we found that YTHDC1 controls HIV-1 protein expression. In particular, we observed that the levels of Env and Vpu proteins were diminished by twofold upon depletion of YTHDC1 in HeLa/LAV and HeLa cells expressing HIV-1, while overexpression of YTHDC1 showed opposite effects in HEK293T cells (Fig. 5 and Additional file 1: Fig. S4). Env and Vpu are encoded by the class of IS bicistronic mRNAs that are up regulated when we knocked down YTHDC1 (Fig. 2). Thus, YTHDC1 appears to down regulate Env/Vpu mRNA abundance while promoting its proteins expression. As we discussed before, m^6^A has been shown to regulate RNA decay and translation of their targets via the recruitment of YTHDF1-3 and YTHDC2 readers [21, 23, 39, 40, 48, 49]. Yet, a similar mechanism has not been described for YTHDC1.

Finally, analysis of viruses produced by HeLa/LAV cells depleted for YTHDC1 suggest that YTHDC1 is required for the efficient release of infectious viral particles in the supernatant and for the processing of Env glycoprotein (Fig. 6). Vpu directly regulates virus release by interfering with the trafficking and the degradation of the cell surface proteins BST-2 and CD4 [48]. For example, by downregulating CD4 in viral producing cells, Vpu was shown to counteract its incorporation in viral particles and favors Env processing [49]. Thus, our results suggest that by regulating the level of expression of Vpu and Env, YTHDC1 could control the infectivity of viral particles.

During the revision process of this article, Tsai and al. reported the binding of YTHDC1 to HIV-1 RNA [50]. Similar to our findings, their results show that YTHDC1 depletion up regulates HIV-1 RNA levels without affecting their nuclear/cytoplasmic distribution. In addition, their study indicates that YTHDC1 regulates alternative splicing of HIV-1 RNAs. However, the analysis of the role of YTHDC1 on viral protein expression gave opposite results. Tsai et al. observed that the main effect of YTHDC1 KD was an increase in Gag expression in 293 T cells infected with HIV-1, whereas we observed a down-regulation of Env and Vpu expression at a post-integration step. Future experiments will be needed to understand if differences in cell types and infection protocols could explain these divergent results.

## Conclusions

Our study shows that the m^6^A methyltransferase complex and the YTHDF2 reader regulate the fate of HIV-1 RNA in HIV-1 producing cells. In addition, this study identified YTHDC1 as a novel reader of HIV-1 transcripts. Similar to what has been described for cellular RNAs, YTHDF2 and YTHDC1 down regulate the abundance of HIV-1 RNAs. Furthermore, we showed that YTHDC1 controls Env and Vpu protein expression and infectivity of HIV-1 particles. Our results highlight a role of the nuclear reader YTHDC1 in the control of post-integration steps of HIV-1 replication.

## Methods

### Cell culture

HeLa, HEK293T, HeLa/LAV (NIH HIV Reagent Program ARP-1301) and TZMbl (NIH HIV Reagent Program ARP-8129) cells were cultivated at 5% CO_2_ and 37 °C in DMEM (Life technologies) supplemented with 10% FBS (Gibco, Life technologies).

### Plasmids and vial stock production

The HIV-1 proviral clone pNL4-3 was developed by M. Martin through the NIH AIDS Reagent Program. Stocks of VSV-G pseudotyped NL4-3 HIV-1 virus were produced by transfecting 4 × 10^6^ HEK293T cells with 3 μg of pNL4-3 and 1 μg of pMD.G (VSV-G) expression vectors using X-tremeGENE HP DNA transfection reagent (Roche), according to the manufacturer’s instructions. Virus containing supernatants were harvested 48 h later and filtered through 0.45 µm filter. Infectious viral titers were measured by infection of HeLa cells with serial dilutions of viral stocks. 24 h.p.i., the percentage of infected cells was determined by FACS analysis by quantifying intracellular capsid (anti-CAp24 antibody, KC57-FITC, Beckman Coulter).

### siRNA treatment and infection of cells

HeLa or HeLa/LAV cells were transfected with 30 nM siRNA (Table 1) using Lipofectamine RNAiMAX (ThermoFisher Scientific) and the reverse transfection protocol according to the manufacturer’s instructions. HeLa cells were plated in 6-well plate at 3 × 10^5^ cells/well the day of siRNA transfection. Cells treated with siRNA targeting METTL3, WTAP, VIRMA were re-seeded at 3 × 10^5^ cells/well in a 6-well plate 3 days after siRNA treatment. 24 h. later cells were infected with VSV-G pseudotyped NL4-3 HIV-1 virus stock at MOI:1. Cells treated with siRNA targeting RBM15, YTHDF1, YTHDF2 and YTHDF3 were not reseeded and directly infected 3 days after siRNA transfection. 24 h.p.i., cells were harvested, washed with PBS1X and RNA was extracted. HeLa/LAV cells were plated in a 6 well plate at 3 × 10^5^ cells/well the day of siRNA transfection. 3 days later, cells and supernatants were harvested. Cells were washed with PBS1X, proteins and RNA were extracted. Media were pooled, filtered and quantified for the HIV-1 CAp24 antigen by an ELISA (Perkin-Elmer).

**Table 1:** siRNA

Gene	Manufacturer	Reference or sequence
Ctrl	Horizon discovery	SMART Pool D-001810-10-50
METTL3	Eurofins	CUGCAAGUAUGUUCACUAUGA
WTAP	Horizon discovery	SMART Pool L-017323-00-0005
VIRMA	Eurofins	GAGGAUGAUCGACGAACAGUA
RBM15	Horizon discovery	SMART Pool L-010854-00-0005
YTHDC1-1	Horizon discovery	J-015332-18-0005
YTHDC1-2	Horizon discovery	J-015332-19-0005
YTHDF1	Eurofins	CCGCGUCUAGUUGUUCAUGAA
YTHDF2	Eurofins	AAGGACGUUCCCAAUAGCCAA
YTHDF3	Eurofins	AUGGAUUAAAUCAGUAUCUAA

### HIV-1 production assays

HEK293T cells were plated in 6-well plate at 3 × 10^5^ cells/well and co-transfected with 1 μg of control plasmid or a GFP-YTHDC1 expression vector and 1 μg of pNL4-3 using Lipofectamine LTX with Plus reagent (ThermoFisher Scientific). HeLa cells treated with 30 nM of siRNA (Ctrl or YTHDC1-1) were transfected the following day with 1 μg of pNL4-3. 4. 48 h. later cells were harvested, washed with PBS1X, then lysed and proteins were analyzed by western blotting.

### RNA extraction

Total RNA from infected HeLa cells or HeLa/LAV cells was extracted with ReliaPrep™ RNA Miniprep Systems (Promega). On-column DNase treatment was performed following provider’s instructions. Samples were treated with a second DNase digestion using TURBO™ DNAse kit (Ambion). The purity and quantity of RNA samples were checked with a NanoDrop 1000 spectrophotometer (Nanodrop Technologies).

### Relative quantification of HIV-1 transcript by RTqPCR

Purified RNAs were reverse transcribed using the High-Capacity cDNA Reverse Transcription Kit (Applied Biosystems) and random primers to avoid bias against long transcripts. Quantification was performed by real-time PCR using LightCycler 480 SYBR Green I Master (Roche) and primer pairs specific for each viral transcript (Table 2). The relative quantification of each viral RNA classes or isoforms was calculated using the ΔΔC_q_ method normalized to the GAPDH reference gene. Total viral RNA primers were used to normalize the relative level of US, IS and MS viral isoforms.

**Table 2:** qPCR primers

Gene	Forward	Reverse	Refs.
Total viral RNA	TTGCTCAATGCCACAGCCAT	TTTGACCACTTGCCACCCAT	[34, 52]
US	TTCTTCAGAGCAGACCAGAGC	GCTGCCAAAGAGTGATCTGA	[52]
IS	GGCGGCGACTGGAAGAAGC	CTATGATTACTATGGACCACAC	[52]
MS	CTGAGCCTGGGAGCTCTCTGGC	CCGCAGATCGTCCCAGATAAG	[53]
Nef2	AGGGGCGGCGACTGGAAGA	GATTGGGAGGTGGGTTGCTTTG	[34, 52]
Rev2	AGGGGCGGCGACTGCCTTA	GATTGGGAGGTGGGTTGCTTTG	[34, 52]
Tat1	AGGGGCGGCGACTGAATTGGGT	GATTGGGAGGTGGGTTGCTTTG	[52]
Env1	AGGGGCGGCGACTGGAAGA	CTATGATTACTATGGACCACAC	[34, 52]
Vpr3	GCGGCGACTGAATCTGCTAT	GCTGCTAGTGCCAAGTACTG	[34, 52]
Vif2	GCGGCGACTGGGACAGCAGAG	GGCACTACTTTTATGTCACT	[34, 52]
preGAPDH	CCACCAACTGCTTAGCACC	CTCCCCACCTTGAAAGGAAAT	
GAPDH	TGCACCACCAACTGCTTAGC	GCATGGACTGTGGTCATGAG	
YTHDF1	CAAGCACACAACCTCCATCTTCG	GTAAGAAACTGGTTCGCCCTCAT	
YTHDF2	TAGCCAGCTACAAGCACACCAC	CAACCGTTGCTGCAGTCTGTGT	
YTHDF3	CGGACAGTGATGCCTACAACAG	TCTGTGAGGTGCGAGGGACTAA	

### Fractionation assay

HeLa/LAV cells were fractionated with Paris kit (ThermoFisher Scientific) using manufacturer’s instructions. RNA extraction for total, cytoplasmic and nuclear fractions were performed using Trizol LS Reagent. Fractions purity was verified by western blot using GAPDH has a cytoplasmic control and LEDGF/p75 has a nuclear control. Nuclear fractions were controlled for enrichment of the pre-mRNA coding for GAPDH (preGAPDH) by RT-qPCR. Cytoplasmic fractions were controlled for enrichment of mitochondrial mRNA MT-CO2 by RT-qPCR. Enrichment of tRNA for each fraction and RNA quality was checked with BioAnalyser Nano 2100 (Agilent) as an additional control of fraction purity. The relative level of each viral RNA classes in cytoplasmic and nuclear fractions was normalized using the GAPDH reference gene.

### RNA co-immunoprecipitation assay

For YTHDC1 co-immunoprecipitation, HeLa/LAV cells were seeded in 10 cm plates at 3 × 10^6^ cells/ plate at the same time as siRNA transfection with siCtrl, siMETTL3 or siRBM15 at 30 nM with Lipofectamine RNAiMAX (Life Technologies) according to previously described protocols [51] and 3 days later, RNA-co-immunoprecipitation was performed (Table 3). For YTHDF2 co-immunoprecipitation, HEK293T cells were seeded in 10 cm plates at 6 × 10^6^ cells/plate 24 h. later, cells were infected with NL4.3 VSVg pseudotyped at MOI:1 for 2 h. in 6 ml of DMEM ± and washed twice with PBS1X. 24 h.p.i. RNA co-immunoprecipitation was performed. RNA was quantified in the immunoprecipitates obtained from formaldehyde-crosslinked cells using a method previously described with some modifications. In brief, siRNA-transfected cells or infected cells were washed with ice-cold PBS and fixed with 0.25% (YTHDC1) or 1% (YTHDF2) formaldehyde in PBS1X for 10 min at 25 °C with gentle manual rocking. Formaldehyde was quenched by adding glycine to a final concentration of 0.25 M and then incubating at 25 °C for 5 min with gentle manual rocking. Fixed cells were washed three times with ice-cold PBS and resuspended in 0.5 ml of radioimmunoprecipitation (RIPA) buffer (50 mM Tris–HCl pH 7.4, 100 mM NaCl, 1% Igepal CA-630, 0.1% SDS, 0.5% sodium deoxycholate, 1 mM DTT) with protease inhibitors (Roche) and RNAsin (400 U/ml, Promega). DNA was sheared by sonication on ice cold bath for 2 s ON, 30 s OFF for 6 cycles with Bioruptor Pico (Diagenode). Lysates were incubated on ice for 5 min, and subjected to DNase I and partial RNaseI digestion for 3 min at 37 °C with mixing 30 μl Turbo™ DNase (Ambion) and 30 μl of RNase I diluted at 1/500 (10.000 U, 100 U/µl, Invitrogen) per 0.5 ml of lysate. After treatment, tubes were immediately transferred into ice and incubated for 5 min. Lysates were then clarified by centrifugation at 21,000*g* at 4 °C for 10 min. 10% of lysates were kept at − 80 °C for RNA Inputs. 250 µl of lysate Protein was subjected to immunoprecipitation in RIPA buffer. Aliquots were kept for western blot analysis. Antibodies targeting YTHDC1 or YTHDF2 (6 μg per 30 μl beads) were first incubated overnight with cells lysate at 4 °C on wheel. Rabbit or mouse IgG antibody was used as a control. The antibody/antigen complexes were then bound to RIPA-buffer-washed Protein A/G magnetic beads (Thermo Fisher). Beads were washed five times with 500 μl wash buffer (50 mM Tris–HCl pH 7.4, 1 M NaCl, 1% Igepal CA-630, 0.1% SDS, 0.5% sodium deoxycholate, 1 mM DTT, 1 M Urea) with protease inhibitors. For each wash, beads are placed on gentle rotation for 10 min at 4 °C. After the last wash, 5 µl of beads are kept for western blot analysis, resuspended in 25 µL of Laemmli 2X and boiled 5’ at 95 °C. The remaining beads are resuspended in 250 μl RNA elution buffer (50 mM Tris–HCl pH 7.4, 5 mM EDTA, 10 mM DTT, 1% SDS). Formaldehyde-induced crosslinks were reversed by incubation at 70 °C for 30 min with mixing. Supernatants were mixed with Trizol LS (Thermo Fisher) and co-immunoprecipitated RNAs were purified according to the manufacturer’s instructions. 20 µg Glycoblue (10 µg/µl, Thermo Fisher) was used to visualize the RNA pellet. Purified inputs RNA and co-immunoprecipitated RNA (IP RNA) were resuspended in 20 µL of UltraPure Distilled water DNase/RNAse free (Invitrogen) and input RNAs were quantified with a NanoDrop 1000 spectrophotometer (Nanodrop Technologies). 1500 ng of input or 17 µL of IP RNA were then reverse-transcribed with random hexamers using High-Capacity cDNA Reverse Transcription Kit (Applied Biosystems) and random primers. RNA levels were detected by qRT–PCR. Relative HIV-1 RNA enrichment was calculated as %of Input using the following equations:

$$2^{\wedge}-({\text{Ct}}_{{{\text{JP RNA}}}}-({\text{Ct}}_{{{\text{Input RNA}}}}-{\text{COMPLEXE.LOG2(Dilution factor}}))*100,$$

where Dilution factor is ((ng of Input RNA/100)/Concentration of Input RNA).

**Table 3:** Antibodies

Name	Reference	
Anti-YTHDC1	Abcam ab264375	Immunoprecipitation
Anti-YTHDC1	Proteintech 14392-1-AP	Western Blot
Anti-YTHDF1	Proteintech 17479-1-AP	Western Blot
Anti-YTHDF2	Millipore ABE542	Immunoprecipitation and Western Blot
Anti-YTHDF3	Proteintech 25537-1-AP	Western Blot
Anti-Gag	NIBSC 90/636	Western Blot
Anti-Env	NIH 2G12	Western Blot
Anti-Vpu	NIH ARP-969	Western Blot
Anti-Nef	NIH 2949	Western Blot
Anti-METTL3	Bethyl A301-567A	Western Blot
Anti-WTAP	Proteintech	Western Blot
Anti-VIRMA	Bethyl A302-124A	Western Blot
Anti-RBM15	Proteintech	Western Blot
Anti-LEDGF/P75	BD Bioscience 611715	Western Blot
Anti-GAPDH	SantaCruz Biotechnology sc-47724	Western Blot
Anti-Mouse HRP	Dako P0260	Western Blot
Anti-Human HRP	Dako P0214	Western Blot
Anti-Rabbit HRP	Dako P0217	Western Blot
IgG Anti-Rabbit	Diagenode C15410206	Immunoprecipitation
IgG Anti-Mouse	Biolegend 400302	Immunoprecipitation

### Infectivity assay

TZM-bl cells were seeded in a 96-well format (10^4^ cells/well) 2 days before infection. Cells were infected with serial dilutions of viral stocks. 48 h.p.i the infectivity of HIV-1 was determined by analysis of firefly luciferase activity measured with CLARIOstar Plus (BMG Labtech). Luciferase activity was normalized by the quantity of CAp24.

### Western blot analysis

Washed cells were lysed for 20 min at 4 °C with radioimmunoprecipitation assay DOC buffer (10 mM Tris pH8, 150 mM NaCl, 1% Triton, 1 mM EDTA, 0.1% Sodium deoxycholate) containing cOmplete protease inhibitor cocktail (Roche). Lysates were spun at 12,000*g* at 4 °C for 10 min, and the supernatant was recovered. The protein concentration was determined using a Bradford protein assay (Bio-Rad), and equal amounts of protein for each sample were mixed with 2XLaemmli buffer (Sigma-Aldrich), boiled for 5 min at 95 °C, and subjected to SDS-PAGE. After protein separation, samples were transferred onto hydrophobic polyvinylidene difluoride (PVDF) membranes, followed by blocking in milk buffer (Tris-buffered saline [TBS] [0.5 M Tris {pH 8.4}, 9% {wt/vol} NaCl], 5% [wt/vol] nonfat dry milk, 0.05% [vol/vol] Tween 20) for 30 min at room temperature (RT). Membranes were incubated overnight at 4 °C with primary antibodies in milk buffer (Table 3). Blots were washed with TBS containing 0.05% (vol/vol) Tween 20 and probed with secondary antibodies in milk buffer for 30 min at RT (Table 3). After washing, protein bands were detected by using Amersham ECL Select Western blotting detection reagent (GE Healthcare). Western blot bands were quantified using the open source image processing package Fiji.

### Supplementary Information

**Additional file 1: Figure S1.** Schematic representation of HIV-1 RNAs detected by RT-qPCR in this study. Organization of the NL4-3 genome and positions of SD and SA sites are indicated. Introns and exons nomenclature are according to [34]. Thick lines represent retained exon and thin lines excised introns. RNA species were named as indicated on the left side according to [34]. Forward (-f) and reverse (-r) primers used in this study are indicated by arrows. Of note, Vpr3 primers designed to detect the Vrp3 IS isoform also amplifies the Vrp1 MS isoform (not shown). However, Vpr3 represents more than 80% of its Vpr transcripts family compare to less than 7% for Vpr1 [34]. **Figure S2.** YTHDF2 depletion increases HIV-1 mRNA transcripts abundance in HeLa infected cells (A) HeLa cells were transfected with a control (Ctrl) or YTHDF1, YTHDF2 and YTHDF3 targeting siRNAs, as indicated. 3 days later, the respective mRNA abundance of each of the 3 readers was measured by RT-qPCR. Data are presented as mean ± S.D. (*n* = 5 for siYTHDF1 and *n* = 3 for siYTHDF2 and siYTHDF3). (B) Cellular proteins knockdown was confirmed by western blot analysis of cell lysates transfected with indicated siRNAs. Note that YTHDF1 was detected on a membrane already probed with the YTHDF2 antibody (°, YTHDF2; *, nonspecific band). (C) 3 days after siRNA transfection, cells were infected with a single round VSVg-pseudotyped HIV-1 virus. 24 h.p.i., the relative abundance of US, IS and MS viral RNAs was monitored by RT-qPCR. Data are presented as mean ± S.D. (*n* = 3). Results are expressed in fold change over the control siRNA (Ctrl). Data are presented as mean ± S.D. (*n* = 3). *P* values were calculated using one-sample t-test (*, p < 0.05, **, p < 0.001). **Figure S3.** YTHDF2 binds preferentially US HIV-1 RNA. (A) HeLa cells were infected with VSVg-pseudotyped HIV-1 and 24 h later YTHDF2 was immunoprecipitated using an antibody against the endogenous protein. IgGs were used as control immunoprecipitation. The specificity of the YTHDF2 immunoprecipitation was confirmed by western blotting and RNA was extracted from input and immunoprecipitated samples. (B) Levels of YTHDF2-associated US, IS and MS viral RNAs were quantified by RT-qPCR. Data are presented as mean ± S.D. (*n* = 3). *P* values were calculated using paired t-test (*, p < 0.05). The p-value for MS RNAs is not significant (p = 0.0821). **Figure S4.** YTHDC1 depletion affects Vpu and Env expression in HeLa and HEK293T producing cells. (A) HeLa cells were transfected with control (Ctrl) or a siRNA targeting YHTDC1 (DC1-1). The following day, cells were transfected with pNL4-3 and 48 h. later cells harvested and cell lysates were analyzed by western blotting for HIV-1 viral proteins Env, Gag and Vpu and Nef. (B) HIV-1 proteins band intensities were quantified by phosphoimaging and normalized to GAPDH (*n* = 3). Fold change of protein levels upon YTHDC1 knockdown over the control are plotted. Data are presented as mean ± S.D. (*n* = 3). *P* values were calculated using one sample t-test (**, p < 0.01).

## Data Availability

Not applicable.

## Abbreviations

gRNA: Genomic RNA
HIV-1: Human immunodeficiency virus type 1
hpi: Hours post-infection
IgG: Immunoglobulin
IgG H Chain: Immunoglobulin heavy chain
IS: Incompletely spliced (IS)
MS: Multiply spliced (MS)
qPCR: Quantitative polymerase chain reaction
RIP: RNA immunoprecipitation
RRE: Rev-response element
S.D.: Standard deviation
US: Unspliced (US)
VSV-G: Vesicular stomatitis virus glycoprotein
