# Correction: YTHDC1 regulates distinct post-integration steps of HIV-1 replication and is important for viral infectivity

**DOI:** 10.1186/s12977-024-00642-1

**Published:** 2024-04-30

**Authors:** Sarah N’Da Konan, Emmanuel Ségéral, Fabienne Bejjani, Maryam Bendoumou, Mélissa Ait Said, Sarah Gallois-Montbrun, Stéphane Emiliani

**Affiliations:** 1 Institut Cochin, INSERM, CNRS, Université de Paris, 75014 Paris, France; 2 Ser vice of Molecular Virology, Department of Molecular Biology, Université Libre de Bruxelles, 6041 Gosselies, Belgium

Following publication of the original article [1], we have been notified that within the Abstract, the word “writer” needs to be replaced with “reader”.

The original article has been corrected.
