# Supplementary figures and images for: YTHDC1 regulates distinct post-integration steps of HIV-1 replication and is important for viral infectivity
Source: Retrovirology. 2022 Jan 31;19:4. doi: 10.1186/s12977-022-00589-1 (PMC8805373; doi:10.1186/s12977-022-00589-1)

Figure S1

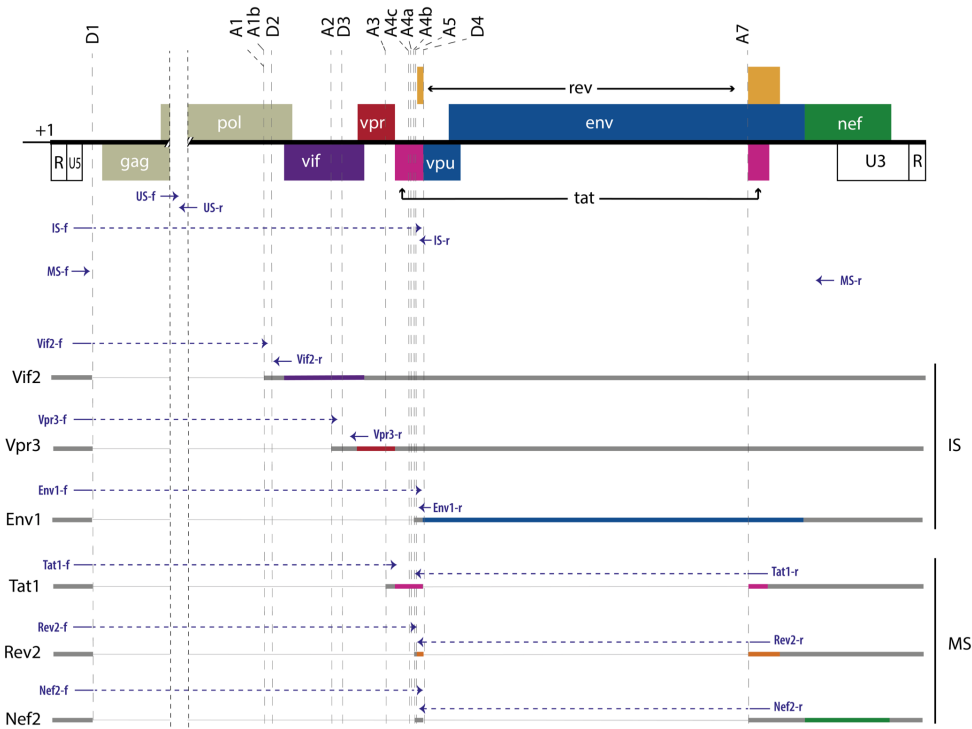

Figure S2

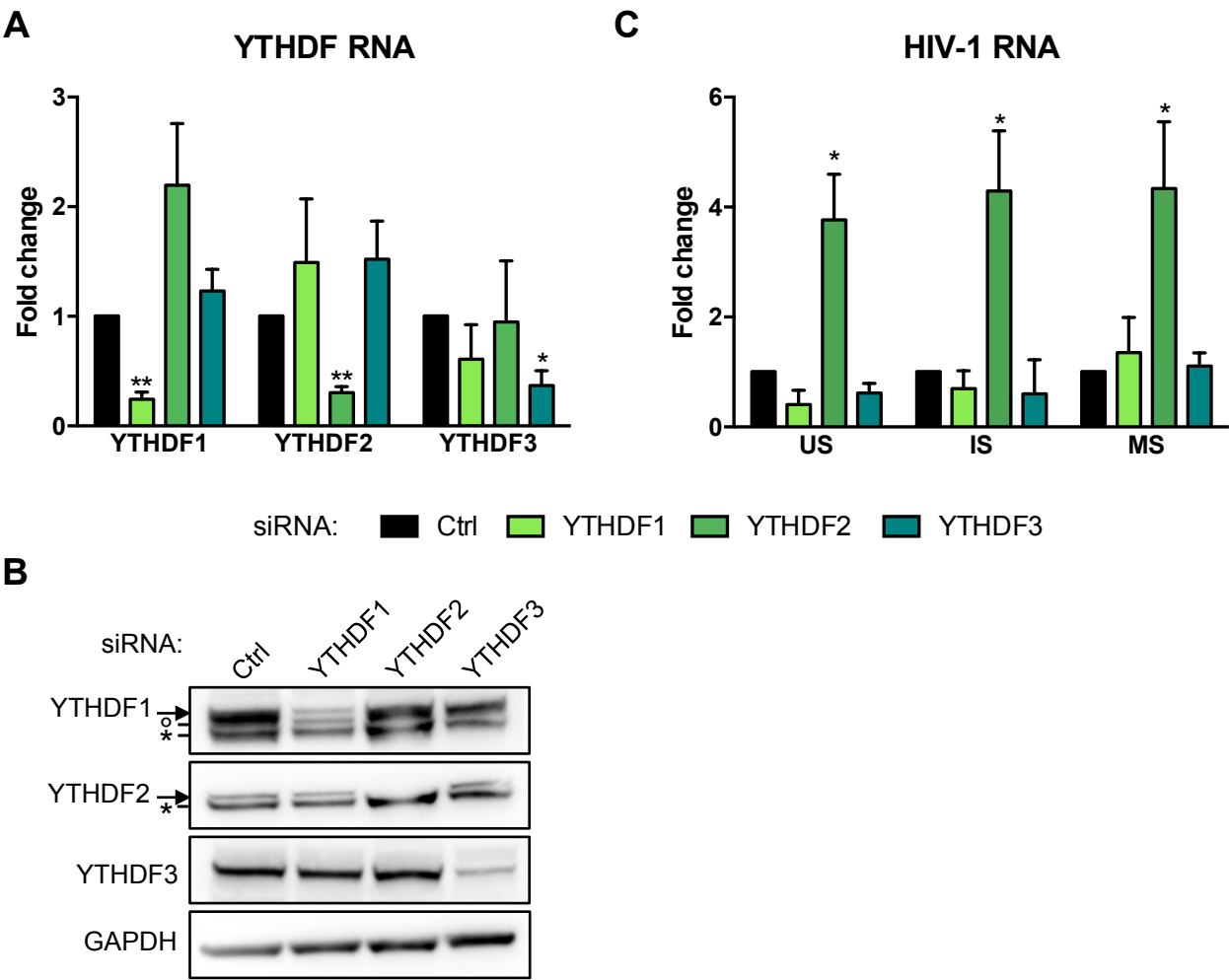

Figure S3

A

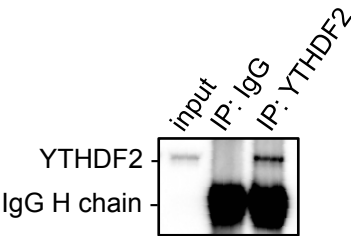

B

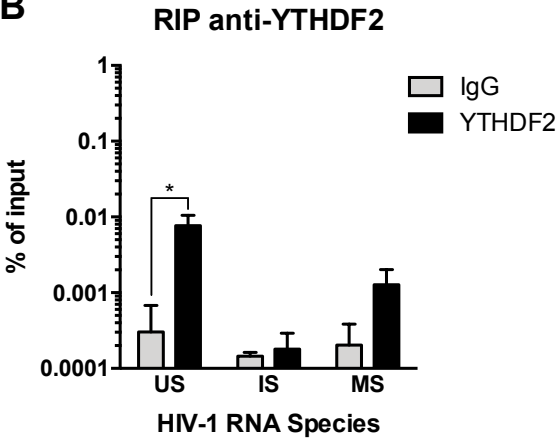

Figure S4

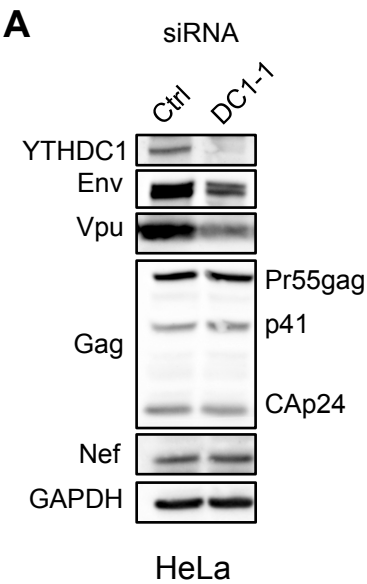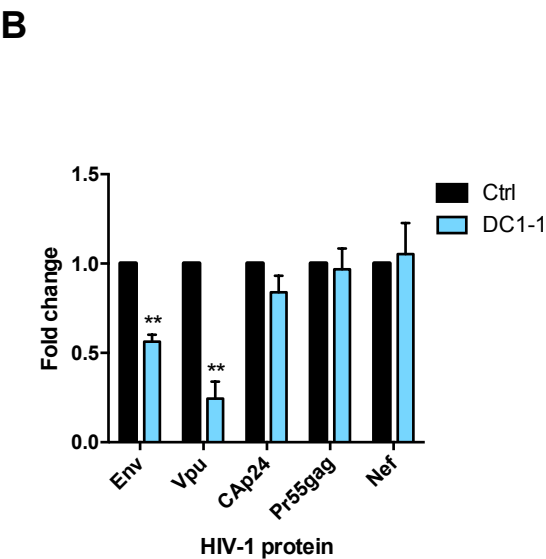

Supplement: Supplementary file 1 — Additional file 1: Figure S1. Schematic representation of HIV-1 RNAs detected by RT-qPCR in this study. Organization of the NL4-3 genome and positions of SD and SA sites are indicated. Introns and exons nomenclature are according to [34]. Thick lines represent retained exon and thin lines excised introns. RNA species were named as indicated on the left side according to [34]. Forward (-f) and reverse (-r) primers used in this study are indicated by arrows. Of note, Vpr3 primers designed to detect the Vrp3 IS isoform also amplifies the Vrp1 MS isoform (not shown). However, Vpr3 represents more than 80% of its Vpr transcripts family compare to less than 7% for Vpr1 [34]. Figure S2. YTHDF2 depletion increases HIV-1 mRNA transcripts abundance in HeLa infected cells (A) HeLa cells were transfected with a control (Ctrl) or YTHDF1, YTHDF2 and YTHDF3 targeting siRNAs, as indicated. 3 days later, the respective mRNA abundance of each of the 3 readers was measured by RT-qPCR. Data are presented as mean ± S.D. (n = 5 for siYTHDF1 and n = 3 for siYTHDF2 and siYTHDF3). (B) Cellular proteins knockdown was confirmed by western blot analysis of cell lysates transfected with indicated siRNAs. Note that YTHDF1 was detected on a membrane already probed with the YTHDF2 antibody (°, YTHDF2; *, nonspecific band). (C) 3 days after siRNA transfection, cells were infected with a single round VSVg-pseudotyped HIV-1 virus. 24 h.p.i., the relative abundance of US, IS and MS viral RNAs was monitored by RT-qPCR. Data are presented as mean ± S.D. (n = 3). Results are expressed in fold change over the control siRNA (Ctrl). Data are presented as mean ± S.D. (n = 3). P values were calculated using one-sample t-test (*, p < 0.05, **, p < 0.001). Figure S3. YTHDF2 binds preferentially US HIV-1 RNA. (A) HeLa cells were infected with VSVg-pseudotyped HIV-1 and 24 h later YTHDF2 was immunoprecipitated using an antibody against the endogenous protein. IgGs were used as control immunoprecipitation. The spe [file 12977_2022_589_MOESM1_ESM.pdf]
